# A global patent dataset of bioeconomy-related inventions

**DOI:** 10.1038/s41597-024-04163-6

**Published:** 2024-11-30

**Authors:** Lukas Kriesch, Sebastian Losacker

**Affiliations:** 1 Department of Geography, Justus Liebig University Giessen, Giessen, Germany; 2 CIRCLE—Center for Innovation Research, Lund University, Lund, Sweden

**Keywords:** Sustainability, Intellectual-property rights, Environmental economics, Climate change

## Abstract

Many governments worldwide have proposed transitioning from a fossil-based economy to a bioeconomy to address climate change, resource depletion, and other environmental concerns. The bioeconomy utilizes renewable biological resources across all sectors and is strongly founded on scientific advances and technological progress. Given that the bioeconomy spans multiple sectors, industries, and technological fields, tracking it is challenging, and both policymakers and researchers lack a comprehensive understanding of the bioeconomy transition’s progress. We aim to solve this problem by providing a dataset on patents, a commonly used indicator to study the development of novel knowledge and technological change, that identifies bioeconomy-related inventions. We leverage the advanced semantic understanding embedded in pre-trained transformer models to identify bioeconomy-related patents based on patent abstracts, and we use a topic modelling approach to identify several coherent technological fields within the corpus of bioeconomy patents. The dataset can be linked to other patent databases and therefore provides rich opportunities to study the technological knowledge base of the bioeconomy.

## Background & Summary

The bioeconomy represents a political vision poised to address critical environmental challenges such as climate change and resource depletion by shifting from an economic system reliant on fossil resources to one centered on renewable biological resources. This approach transforms the economy by utilizing biological resources and knowledge to produce goods, services, and energy across all sectors, thereby diminishing reliance on non-renewable resources^1,2^. Currently, this idea has been embraced (in very similar ways) by over 60 countries worldwide, each offering policy strategies to facilitate the bioeconomy transition^3,4^. The current implementation of the bioeconomy in global policy programs is significantly influenced by the concept of the Knowledge-Based Bio-Economy introduced by the European Commission in 2005. Back then, the shift toward a bioeconomy was envisioned primarily through the lens of novel knowledge and advances in technology and science. Although this technology-centric perspective of the bioeconomy has expanded over time, knowledge and innovation continue to be regarded as essential pillars of the bioeconomy^4–6^.

To evaluate and support the bioeconomy, scholars and policymakers rely on accurate measurement and monitoring of bio-based activities. The progress of the bioeconomy has been empirically measured, for example, through the volume of processed biomass, through the number of bioeconomy firms or through estimations of employment or value added related to the bioeconomy^7–10^. In line with the role that innovation and technological change play within the bioeconomy, as we have argued above, several attempts have also been made to track knowledge and innovation activities that contribute to the bioeconomy transition^11–14^. Arguably, one of the most commonly used indicators to study innovation and knowledge development in that respect, and on a very general level, is patent data.

However, using patent data to measure knowledge development and innovation has certain limitations that need to be acknowledged. Most importantly, not every patented invention reaches the market, remaining an invention rather than becoming an innovation. Simultaneously, not all inventions or innovations are patented, either due to strategic decisions by the inventor or the limitations of patent systems in protecting intellectual property in certain industries (e.g., software, services, or creative sectors). As a result, the propensity to patent varies between industries. Patents primarily capture technological innovations, while many other forms of innovation, such as social innovations, organizational innovations, and business model innovations, cannot be tracked using patent data^15–18^.

Despite the known shortcomings of patent data as an indicator of knowledge development and innovation activity, it remains one of the most accessible and important data sources. Patent data covers many technological domains, is available globally, and spans long time periods. Additionally, it can be easily sourced through platforms like PATSTAT^19^. Patent data provides information on the inventor and applicant, their locations, and time (e.g., date of application). It also includes several legal details such as licensing and infringement, as well as content of the patented invention (e.g., title, patent claims, abstract, drawings). Patents can be classified according to technological classification systems, such as the Cooperative Patent Classification (CPC), and they link to prior inventions through citations to other patents or scientific literature.

Using patent data to understand new technological trends and changes in a specific domain is commonly referred to as ‘patent landscaping’. Identifying patents relevant to the bioeconomy is a significant challenge in patent landscaping. Research on patent landscapes often deals with the problem of locating patents related to specific topics or technologies. Conventional methods usually use technology classifications, keywords, citations, or a combination of these elements to define a particular topic within the vast patent landscape. Limitations associated with these traditional techniques have been studied in depth in scientific discussions^15–18,20^. The use of keywords is vulnerable to the inherent variability of language, where a single concept may be formulated using a variety of terminologies, and a single term may have multiple meanings. The multidimensional nature of the bioeconomy, which encompasses a wide range of sectors, further complicates the identification of appropriate keywords and technology classifications. A patent could pertain to the bioeconomy but may be classified within a technology class not commonly associated with it. Furthermore, it has been observed that errors in technology classification occur at patent offices due to mistakes, misclassifications, or misprints, as both automated and human-generated classifications are utilized. It has been noted that some applicants, such as firms, deliberately seek misclassifications for strategic reasons to conceal the true application of an invention^21^. Rule-based approaches (i.e., the use of technology classes and/or keywords) are thus prone to numerous errors, often rendering them unreliable for accurately identifying patents belonging to a specific technological field. Recent advances in patent landscaping have utilised the content of patent abstracts, using modern machine learning methods to accurately define specific technologies or topics within the patent database^22^.

In this study, we leverage the advanced semantic understanding embedded in pre-trained transformer models, which have shown superior performance in the domain of patent classification. These models have demonstrated substantial advantages over traditional keyword-based approaches and other machine learning architectures, such as convolutional neural networks and multi-layer perceptrons, particularly in achieving higher precision, recall, and F1-scores across various technological domains^22^. Transformer models are particularly adept at capturing complex language patterns and contextual nuances, rendering them well-suited for accurately classifying technologies. By leveraging these natural language processing (NLP) capabilities, it is possible to tailor the models specifically for identifying bioeconomy patents. This fine-tuning is achieved with minimal reliance on extensive annotated datasets, as discussed by Ruder *et al*.^23^. A crucial aspect of this adaptation process is the strategic selection of data points, which is essential not only for refining the model to the specific task but also for assessing its predictive performance. The importance of this step is particularly emphasized in patent landscaping, where the precision and variety of the training data are critical to accurately capture the targeted field^24^.

## Methods

### Stage 1: data acquisition

We used data from the PATSTAT 2022 Spring Edition^25^ and selected English patent abstracts. The dataset consists of 67 million unique English patents. By limiting our analysis to these documents, we ensured a consistent linguistic framework for subsequent analyses. Given that we only include patent applications with English abstracts, we exclude those submitted to national patent offices where the abstracts are in other languages. For patent families—i.e., multiple patent applications for the same invention filed with different national patent offices and potentially in different languages—users of our dataset^26^ can link patents with abstracts in other languages to the corresponding patent family. When the same invention is patented at different offices with English abstracts, these patents are captured and processed multiple times. Users can then combine these patents within the patent family. This approach provides flexibility, allowing users to choose whether to analyze data at the individual patent level or the patent family level, depending on their interest and research goals.

### Stage 2: Selection and annotation of high-quality training data

We started the training procedure with the annotation of a foundational set of patent abstracts, sourced from bioeconomy-related technology categories as identified by Frietsch *et al*.^27^. We thus begin our classification approach by using technology codes that are likely to capture bioeconomy-related patents. However, to avoid the limitations of relying solely on these technology codes (see above), we do not consider them in the subsequent steps of the analysis. To contrast, for the control group, we randomly selected patent abstracts from the broader patent corpus and annotated them manually with a group of five human annotators. In our annotation guidelines, we adopt the definition of the bioeconomy provided by the European Union. According to this definition, the bioeconomy is primarily defined as “the production of renewable biological resources and their transformation, along with waste streams, into value-added products like food, feed, bio-based products, and bioenergy” (EC 2012, p. 9)^28^. Given this definition, the bioeconomy encompasses novel bio-based products, services and processes, as well as the processes and products used to manufacture and produce them. This broader definition encompasses a wide range of sectors and also includes inventions that are not bio-based themselves, but are important for the production of bio-based products and processes.

**Fig. 1:**
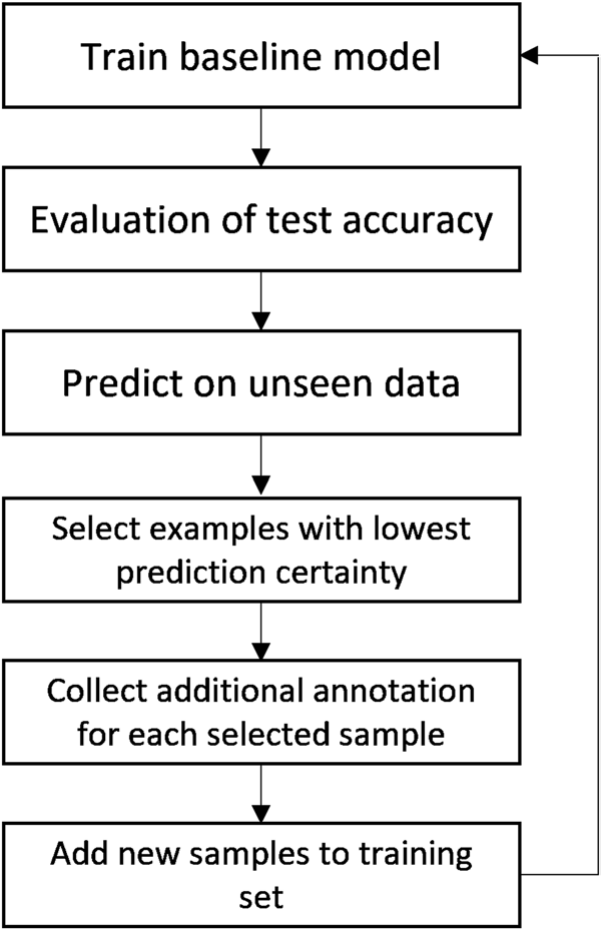
Active learning process.

This initial dataset served to train a baseline model (mixedbread-ai/mxbai-embed-large-v1)^29^ using a framework for few-shot fine-tuning pre-trained Sentence Transformers models^30,31^. To enhance this model, we employed an active learning strategy^32^. This involved using the baseline model to assess a random selection of patent abstracts, iteratively focusing on those with the lowest levels of prediction certainty. Figure 1 depicts the active learning process.

**Fig. 2:**
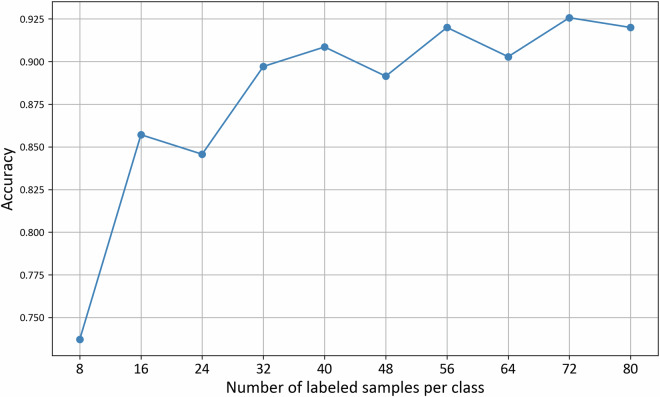
Learning curve of the final model.

We collected additional annotations for each selected sample and added these to the training dataset. This approach mitigates potential biases from the initial selection of training data, as the active learning strategy progressively incorporates more diverse and challenging samples beyond the original seed data. We trained subsequent models on this progressively enriched dataset, repeating the cycle until no significant improvement in accuracy was observed. This iterative approach to annotation not only conserves time and resources but also selectively targets data points that are most likely to enhance the model’s predictive accuracy. Following the creation of a comprehensive and representative dataset comprising 350 manually annotated samples, we conducted a series of evaluations on various pretrained large language models (see technical validation). We used 50% of the data for training and 50% for testing. Figure 2 depicts the learning curve of the model evaluated on the test set.

To assess the consistency and agreement among human annotators, we conducted intercoder reliability checks on a subset of 200 edge cases selected from both the training and test datasets. A Krippendorff’s alpha coefficient of 0.797 indicates a substantial level of agreement among the annotators regarding the classification of bioeconomy-related patents, validating the robustness of our annotation process and the reliability of the training data^33^.

### Stage 3:Predicting bioeconomy probability across the full patent dataset

In the third stage of our analysis, we applied our trained text classification model to predict the probability of each patent belonging to the bioeconomy domain. Patents with a predicted probability of more than 50% were classified as bioeconomy-related. Our model successfully identified 5,639,054 (8.32%) patents as bioeconomy-related, highlighting the significant contribution of bio-based innovation within the patent landscape.

## Data Description

Our analysis revealed that bioeconomy-related patents span across a diverse array of technological domains, as we find bioeconomy-related patents across 660 out of 672 classes at the four-digit level of the CPC system. This broad coverage underscores the multifaceted nature of the bioeconomy, encompassing various sectors and disciplines. Table 1 illustrates these classes, highlighting those with the largest proportion of bioeconomy patents.

**Table 1:** Top ten CPC classes with highest share among identified bioeconomy related patents.

CPC	Description of CPC Class	Count Bioeconomy	Share Among Bioeconomy	Total Count	Total share
A61K	Preparations for medical, dental or toiletry purposes	2,647,960	13.98%	9,169,135	3.92%
C12N	Microorganisms or enzymes; compositions thereof; propagating, preserving, or maintaining microorganisms; mutation or genetic engineering; culture media	1,419,440	7.49%	2,146,634	0.92%
A61P	Specific therapeutic activity of chemical compounds or medicinal preparations	1,288,110	6.8%	7,760,426	3.32%
C02F	Treatment of water, waste water, sewage, or sludge	1,034,660	5.46%	1,476,213	0.63%
A23L	Foods, foodstuffs, or non-alcoholic beverages, not covered by subclasses A21D or A23B-A23J; their preparation or treatment, e.g. cooking, modification of nutritive qualities, physical treatment (shaping or working, not fully covered by this subclass, A23P); preservation of foods or foodstuffs, in general	718,651	3.8%	931,437	0.4%
C07K	Peptides	620,067	3.27%	2,066,851	0.88%
G01N	Investigating or analysing materials by determining their chemical or physical properties	588,334	3.11%	3,492,002	1.49%
A01N	Preservation of bodies of humans or animals or plants or parts thereof	538,706	2.85%	698,756	0.3%
B01D	Separation	421,493	2.23%	2,538,268	1.08%
C12Q	Measuring or testing processes involving enzymes, nucleic acids or microorganisms (immunoassay G01N33/53); compositions or test papers therefor; processes of preparing such compositions; condition-responsive control in microbiological or enzymological processes	335,217	1.77%	790,137	0.34%

Notably, classes such as “Preparations for medical, dental or toiletry purposes” (CPC class A61K) and “Microorganisms or enzymes; compositions thereof” (CPC class C12N) stand out with substantial shares of bioeconomy patents. These findings highlight the diverse applications of bio-based technologies in areas ranging from pharmaceuticals to environmental remediation.

Moreover, Table 2 highlights CPC classes with exceptionally high proportions of bioeconomy patents, underscoring specialized domains within the bioeconomy landscape. For instance, CPC class A01H, which pertains to new plants or non-transgenic processes, exhibits a high bioeconomy share of 99.21%. Similarly, classes such as C05F, C05G, and C05B, which deal with various types of fertilizers, demonstrate substantial bioeconomy representation, emphasizing the significance of agricultural and food-related innovations in the bioeconomy domain.

**Table 2:** Top ten CPC classes with highest density of bioeconomy related patents.

CPC	Description	Total count	Count bioeconomy	Share
A01H	New plants or non-transgenic processes for obtaining them; plant reproduction by tissue culture techniques	143,833	142,701	99.21%
C05F	Organic fertilisers not covered by subclasses C05B, C05C	69,479	67,086	96.56%
C05G	Mixtures of fertilisers covered individually by different subclasses of class C05	93,155	89,567	96.15%
C05B	Phosphatic fertilisers	33,410	31,681	94.89%
C12J	Vinegar; preparation or purification thereof	4,776	4,484	93.89%
C12R	Indexing scheme associated with subclasses C12C - C12Q, relating to microorganisms	53,109	49,582	93.36%
A23Y	Indexing scheme relating to lactic or propionic acid bacteria used in foodstuffs or food preparation	38,008	35,198	92.61%
A23K	Fodder	319,987	295,492	92.35%
C05D	Inorganic fertilisers not covered by subclasses C05B, C05C	25,332	23,276	91.88%
A23J	Protein compositions for foodstuffs; working-up proteins for foodstuffs; phosphatide compositions for foodstuffs	37,086	33,185	89.48%

In summary, our analysis provides comprehensive insights into the distribution and prevalence of bioeconomy patents across diverse technological domains, highlighting the breadth and depth of bio-based innovation within the global patent landscape.

While traditional classification methods, such as CPC codes, provide valuable insights into the technological domains of patents, they may not fully capture the diverse thematic areas and cross-disciplinary relationships within the bioeconomy domain. To address this limitation, we use topic modeling to identify hidden semantic structures and thematic clusters within the corpus of bioeconomy patents.

**Fig. 3:**
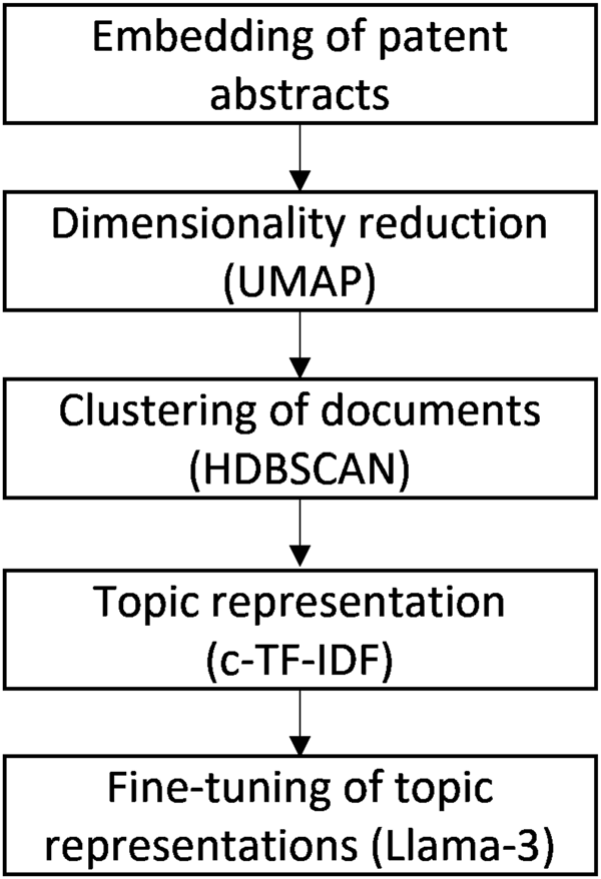
Topic modelling process.

In our approach, we utilize the BERTopic Framework^34^ to derive these thematic clusters. This modular framework streamlines the topic modeling process through five key steps, as illustrated in Fig. 3.

Firstly, we employ the “mixedbread-ai/mxbai-embed-large-v1” sentence transformer model to convert patent abstracts into numerical vectors, capturing their semantic representations. Subsequently, we use Uniform Manifold Approximation and Projection (UMAP) for dimensionality reduction, refining the vectors to facilitate efficient clustering in the subsequent steps.

The third step involves clustering the dimension-reduced vectors using Hierarchical Density-Based Spatial Clustering of Applications with Noise (HDBSCAN), a robust technique that adapts well to varying cluster shapes and densities. An important parameter for the clustering is the minimal cluster size, which strongly affects the number of created topics and the number of outliers. A smaller cluster size leads to more clusters and fewer outliers, while a larger cluster size produces fewer clusters but more outliers. Therefore, choosing cluster sizes is a trade-off between the number of outliers and the number of clusters^35,36^. We set the cluster size to 0.01% of the document corpus size to achieve an explainable number of topics.

The fourth step is topic generation with a class-based version of the term frequency – inverse document frequency measure (c-TF-IDF). c-TF-IDF is an adaptation of TF-IDF^37^, which is designed to generate representative terms for each cluster. Finally, to translate these descriptions into topic labels, we leverage “Meta-Llama-3-8B”, an open-source large language model. This last step enhances interpretability by providing human-readable labels for the identified thematic clusters. To reduce the number of outliers, we used an outlier reduction strategy. This approach involves merging outlier documents with their nearest existing topic by finding the most frequent topic in each outlier document. This method helps in minimizing the number of unassigned documents, thereby enhancing the coherence and interpretability of the identified topics.

Overall, we identified 98 topics. Table 3 shows the ten largest topics identified through our topic modeling process applied to the bioeconomy patent dataset.

**Table 3:** Top ten largest topics in the bioeconomy patent dataset.

Topic representation	Count	Share	Topic label
organic fertilizer, fertilizer, fertilizers, soil, preparation method, planting method, cultivation, nutrient, organic, planting	355,735	7.0%	Organic Plant Cultivation Methods
feeding device, feeding trough, animal husbandry, feeder, livestock, husbandry, cattle, breeding, animal, mechanism	217,539	4.3%	Animal Husbandry Feeding Mechanisms
food processor, food processing, food packaging, food product, cooker, container, processing, machine, mechanism, refrigerator	199,567	4.0%	Food Processing and Packaging Technology
water purifier, water purifying, water purification, purifier, membrane filter, purified water, reverse osmosis, water dispenser, filtration, water treatment	146,776	2.9%	Water Purification and Filtration Systems
sewage treatment, sewage, treatment tank, treatment equipment, domestic sewage, field sewage, sedimentation tank, wastewater, treatment device, filter tank	129,003	2.6%	Sewage Treatment and Management Systems
seasoning, soy sauce, preparation method, fish meat, cooking, soybean paste, flavor, spice, pork, meat	125,227	2.5%	Food Preparation and Seasoning Methods
recombinant protein, gene expression, recombinant, cdna, fusion protein, polypeptides, heterologous, polynucleotide, expression vectors, plasmid	125,101	2.5%	Biotechnology and Gene Expression
chinese medicinal, chinese medicines, chinese medicine, chinese herbal, medicinal composition, medicine composition, medicinal materials, traditional chinese	121,888	2.4%	Traditional Chinese Medicine Compositions
fish tank, aquarium, water tank, fish, tank, fishes, aquaculture, aquatic products, pond’, net cage	118,462	2.3%	Aquatic Farming and Aquaculture Methods
pig feed, feed additive, feed prepared, compound feed, chinese herbal, feeding, pigs, feed, fodder, pig	111,356	2.2%	Animal Feed and Nutrition

Each topic represents a distinct thematic cluster within the domain, characterized by a representative label, the count of patents associated with that topic, and its share of the total corpus. The topics encompass a wide range of innovations and research areas within the bioeconomy domain, spanning agriculture, biotechnology, environmental science and food technology.

**Fig. 4:**
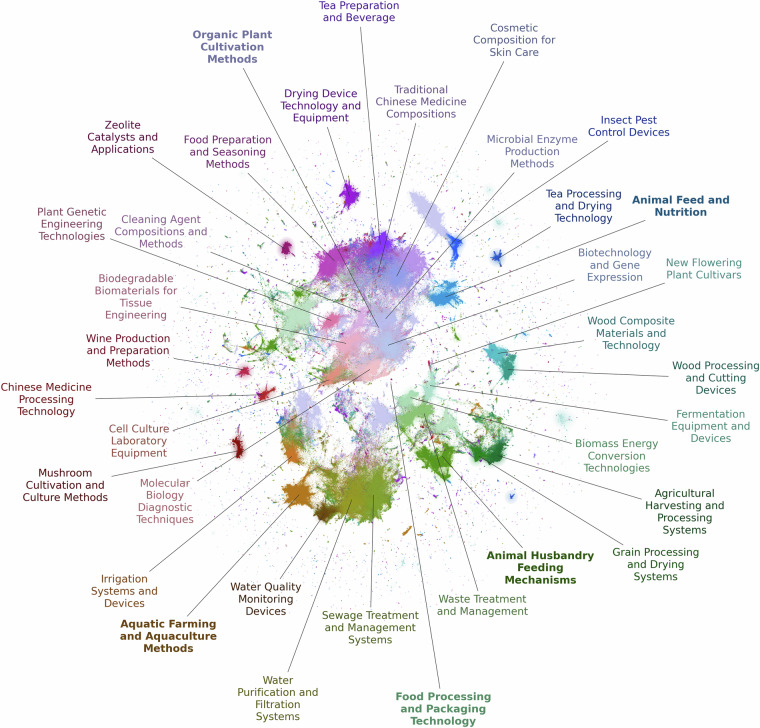
Datamap of bioeconomy topics.

Figure 4 displays the 36 largest topics and their semantic proximity.

## Data Records

The data is stored in a repository hosted by the Open Science Framework^26^. In the completed dataset, every row corresponds to the data of a single patent, including its likelihood of being classified under the bioeconomy category. This likelihood is quantified by the “prob_bioeconomy” value, where a higher number indicates a greater probability that the model classifies the patent as part of the bioeconomy. The topic number assigned to each patent is shown by the variable “topic”. We also share a dataset for matching the topic number with the topic descriptions and labels. The patent dataset columns are explained below:

1. appln_id: Application ID of the patent
2. prob_bioeconomy: This column quantifies the likelihood of a patent belonging to the bioeconomy domain. A higher value indicates a greater probability that the model classifies the patent as part of the bioeconomy. This probability is provided for all 67 million patents in the dataset.
3. topic: This number corresponds to the thematic cluster or topic derived from the topic modeling process. Topics are available for patents with a prob_bioeconomy greater than 0.5. For patents with a prob_bioeconomy less than 0.5, indicating a lower likelihood of belonging to the bioeconomy domain, the topic value is designated as “NA”, signifying that no topic has been assigned.

## Technical Validation

To validate the performance of our text classification model, we compared three leading open-source models: “mixedbread-ai/mxbai-embed-large-v1”^29^, “BAAI/bge-large-en-v1.5”^38^ and the “PatentSBERTa”^39^ model, which was specifically pretrained on patent data. This comparison aimed to assess each model’s accuracy, precision, recall, and F1-score in classifying bioeconomy-related patents. We conducted 5-fold cross-validation to ensure robustness and generalizability of the results. Table 4 summarizes the performance metrics obtained from this evaluation.

**Table 4:** Results of model comparison.

Model	Accuracy mean ± std)	Precision (mean ± std)	Recall (mean ± std)	F1-Score (mean ± std)
AI-Growth-Lab/PatentSBERTa	92.28% ± 0.025	88.45% ± 0.07	**91.90% **±** 0.046**	89.91% ± 0.031
mixedbread-ai/mxbai-embed-large-v1	**94.28% **±** 0.022**	**93.89% **±** 0.061**	90.96% ± 0.02	**92.26% **±** 0.027**
BAAI/bge-large-en-v1.5	93.43% ± 0.012	93.63% ± 0.012	88.60% ± 0.012	91.10% ± 0.06

Our comparative analysis revealed that while all models demonstrated high efficacy in classifying bioeconomy patents, the “mixedbread-ai/mxbai-embed-large-v1” model exhibited the most balanced and superior performance across most metrics. Consequently, we selected this model for further application due to its robustness and overall accuracy.

**Fig. 5:**
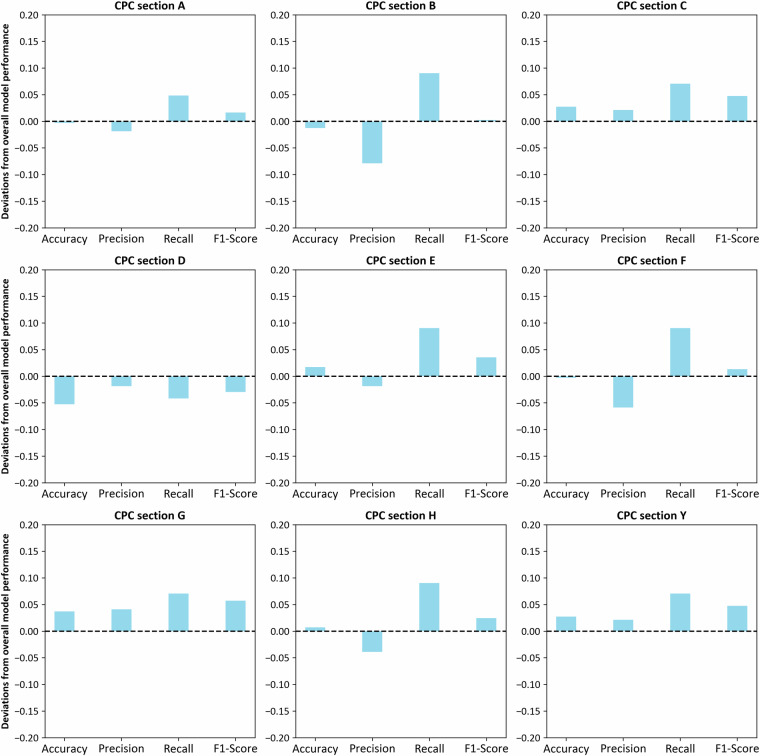
Deviations in model performance across CPC sections.

To evaluate the model’s performance, we validated it using a balanced sample of 900 patents, stratified evenly across nine CPC sections. Specifically, 100 patents were selected from each section, consisting of 50 bioeconomy-related and 50 non-bioeconomy-related predicted cases, and the model’s predictions were manually verified. Figure 5 presents the deviations observed in each section, comparing them to the overall metrics from the active learning training dataset. This comparison offers a more detailed insight into the model’s performance across different segments of the patent corpus. For CPC class D, the model achieves an accuracy of 89%, while for class Y, it achieves 97%. The performance metrics for the majority of CPC sections exceed the overall metrics, with a slight decline in performance observed only in CPC section D (Textiles; Paper).Overall, the consistency of these performance metrics across the nine classes demonstrates that the model is robust and capable of generalizing well to various technological domains. There are no substantial deviations in the performance metrics across the CPC classes, suggesting that the model’s classification capabilities are stable and reliable across different types of patents.

**Fig. 6:**
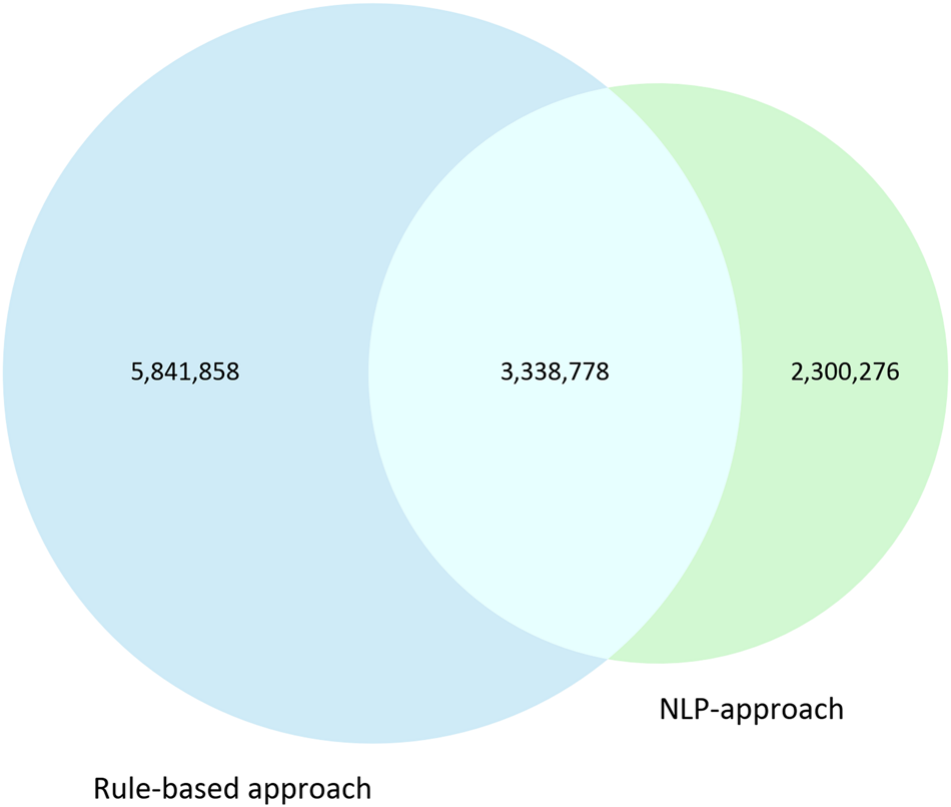
Comparison between rule-based approach and NLP-approach for classifying bioeconomy patents.

Additionally, we conducted a comparison of our bioeconomy classification approach with classification methods utilizing International Patent Classification (IPC) codes, as outlined by Frietsch *et al*.^27^. Among the 5,639,054 patents identified through our study, 3,338,778 (59.2%) patents were also identified using the IPC code search strategy. Conversely, 5,841,858 patents identified via IPC codes were not present in our dataset. Figure 6 displays the overlap and the distinct number of patents identified by each search strategy.

To gain a better understanding of the patents not captured by our identification strategy, we examined the IPC classes of those cases. Table 5 provides an overview of the largest IPC classes among the patents identified via IPC codes but not present in our dataset.

**Table 5:** Largest IPC classes of patents unique to IPC code search strategy.

IPC Code	Description	Count	Share
B29C	Shaping or joining of plastics; shaping of material in a plastic state, not otherwise provided for; after-treatment of the shaped products, e.g. repairing	2,124,806	16.95%
B41J	Typewriters; selective printing mechanisms, i.e. mechanisms printing otherwise than from a forme; correction of typographical errors	1,120,060	8.94%
C07K	Peptides	736,910	5.88%
C12N	Microorganisms or enzymes; compositions thereof	634,639	5.06%
C08J	Working-up; general processes of compounding; after-treatment not covered by subclasses C08B, C08C, C08F, C08G or C08H	513,295	4.09%
A47J	Kitchen equipment; coffee mills; spice mills; apparatus for making beverages	472,178	3.77%
E02D	Foundations; excavations; embankments; underground or underwater structures	409,068	3.26%
D06M	Treatment, not provided for elsewhere in class D06, of fibres, threads, yarns, fabrics, feathers or fibrous goods made from such materials	398,786	3.18%
B41M	Printing, duplicating, marking, or copying processes; colour printing	346,542	2.77%
C12Q	Measuring or testing processes involving enzymes or microorganisms; compositions or test papers therefor; processes of preparing such compositions; condition-responsive control in microbiological or enzymological processes	317,526	2.53%

In line with Table 5, we also examine the IPC classes of patents that are unique to our classification approach. Table 6 displays the top ten IPC classes with the highest share among these patents. Both classification approaches have inherent strengths and weaknesses that account for the varied outcomes observed. Our NLP approach relies heavily on the information provided within patent abstracts. However, this reliance introduces vulnerabilities, as some abstracts may be inaccurately formulated or lack explicit mention of bio-related products or processes. Additionally, we found a small fraction of patent abstracts (0.063%) with less than ten words. Many of these abstracts are incomplete or shortened formulations, leading to their exclusion from classification under the bioeconomy domain. Conversely, the lack of specificity inherent in class codes results in the categorisation of patents based on technological and functional principles that may not fully align with the multidisciplinary nature of the bioeconomy. Consequently, patents relevant to the bioeconomy might be dispersed across multiple classes, thereby complicating accurate identification through class codes alone. Furthermore, the predefined categories of class codes may not comprehensively cover all aspects of the bioeconomy, leading to the exclusion of patents that contribute to this domain but do not fit neatly into existing class codes. The subjectivity inherent in classifying patents using class codes further complicates the issue, introducing inconsistencies and errors in the classification process. Furthermore, the use of class codes may result in a high number of false positives, with patents being incorrectly identified as bioeconomy-related due to the broad scope of some class codes. Additionally, the classification system may be unable to capture emerging trends and innovations within the bioeconomy, as there may be a lag in updating classifications to reflect these developments. Despite the points mentioned above, users of our dataset^26^ can tailor their analysis by combining our data with rule-based approaches, such as filtering for bioeconomy-related patents identified in our dataset within a specific CPC class.

**Table 6:** Largest IPC classes of patents unique to NLP search strategy.

IPC Code	Description	Count	Share
A61K	Preparations for medical, dental, or toilet purposes	1,212,029	17.21%
C02F	Treatment of water, waste water, sewage, or sludge	863,629	12.26%
A61P	Specific therapeutic activity of chemical compounds or medicinal preparations	523,812	7.44%
B01D	Separation	406,595	5.77%
G01N	Investigating or analysing materials by determining their chemical or physical properties	333,526	4.74%
F26B	Drying solid materials or objects by removing liquid therefrom	226,862	3.22%
B01J	Chemical or physical processes, e.g. catalysis, colloid chemistry; their relevant apparatus	160,974	2.29%
B65D	Containers for storage or transport of articles or materials, e.g. bags, barrels, bottles, boxes, cans, cartons, crates, drums, jars, tanks, hoppers, forwarding containers; accessories, closures, or fittings therefor; packaging elements; packages	147,903	2.10%
C07D	Heterocyclic compounds	138,354	1.96%
A61L	Methods or apparatus for sterilising materials or objects in general; disinfection, sterilisation, or deodorisation of air; chemical aspects of bandages, dressings, absorbent pads, or surgical articles; materials for bandages, dressings, absorbent pads, or surgical articles	132,898	1.89%

## Usage Notes

The dataset^26^ can be linked to the overall PATSTAT dataset and to other patent datasets via the appln_id^19,40,41^. Some patent abstracts sourced from PATSTAT are incomplete and therefore not classified as bioeconomy related with our approach. We suggest for these cases (e.g., for patent abstracts with less than ten words) to use a combination of our classification and CPC codes.

## Data Availability

All Python code produced for this project can be accessed on: https://github.com/LukasKriesch/BE_patents.
